# Prediction of protein-protein interaction sites in intrinsically disordered proteins

**DOI:** 10.3389/fmolb.2022.985022

**Published:** 2022-09-30

**Authors:** Ranran Chen, Xinlu Li, Yaqing Yang, Xixi Song, Cheng Wang, Dongdong Qiao

**Affiliations:** ^1^ Department of Biostatistics, School of Public Health, Cheeloo College of Medicine, Shandong University, Jinan, China; ^2^ National Institute of Health Data Science of China, Shandong University, Jinan, China; ^3^ Shandong Mental Health Center, Shandong University, Jinan, China

**Keywords:** intrinsically disordered protein (IDP), protein interaction sites prediction, machine learning, ML, protein functions, protein sequence

## Abstract

Intrinsically disordered proteins (IDPs) participate in many biological processes by interacting with other proteins, including the regulation of transcription, translation, and the cell cycle. With the increasing amount of disorder sequence data available, it is thus crucial to identify the IDP binding sites for functional annotation of these proteins. Over the decades, many computational approaches have been developed to predict protein-protein binding sites of IDP (IDP-PPIS) based on protein sequence information. Moreover, there are new IDP-PPIS predictors developed every year with the rapid development of artificial intelligence. It is thus necessary to provide an up-to-date overview of these methods in this field. In this paper, we collected 30 representative predictors published recently and summarized the databases, features and algorithms. We described the procedure how the features were generated based on public data and used for the prediction of IDP-PPIS, along with the methods to generate the feature representations. All the predictors were divided into three categories: scoring functions, machine learning-based prediction, and consensus approaches. For each category, we described the details of algorithms and their performances. Hopefully, our manuscript will not only provide a full picture of the status quo of IDP binding prediction, but also a guide for selecting different methods. More importantly, it will shed light on the inspirations for future development trends and principles.

## 1 Introduction

With the rapid development in the protein field, there are increasingly number of intrinsically disordered proteins (IDPs) and intrinsically disordered protein regions (IDRs) identified in viruses, bacteria, archaea, and eukaryotes (Dubreuil et al., 2019).

IDPs lack stable tertiary structure under physiological conditions and are highly flexible compared with globular proteins (Uversky et al., 2008). Therefore, when interacting with other proteins, IDP can participate in various physiological processes (Uversky et al., 2005) including cell signal transduction and regulation through conformational changes. IDPs are also widely involved in diseases. For example, type 1 susceptible protein (BRCA1) involved in the occurrence of breast cancer participates in the interaction mainly through disordered regions (Uversky et al., 2008), and α -synuclein folds from disordered state in acidic or high temperature environment, which leads to neurodegenerative diseases (Uversky et al., 2008).

IDPs take part in protein-protein interactions through one-to-many or many-to-one mode (Uversky et al., 2008). Studies found that the protein-protein interaction in which IDP participates is often achieved through coupled folding and binding (Dyson and Wright, 2002). For example, CREB protein forms a spiral structure by binding CBP protein (Radhakrishnan et al., 1997), and the binding of p53 protein with MDM2 protein leads to the protein folding from coil to spiral (Kussie et al., 1996). It has been found that the binding of IDP with corresponding proteins will lead to the transition from disorder to order. For instance, the disordered regions of E-cadherin turn to order format after interacting with β-catenin (Dyson and Wright, 2002); the disordered protein DFF45 interacts with DFF40 to transform into an ordered state (Zhou et al., 2001); the Furin-like cleavage site exists at the S1/S2 junction of the SARS-CoV-2 Spike (FLCS_Spike_), and the “disorder-to-order transition” of Spike-Furin complex has been found (Roy et al., 2022). In addition, some ordered proteins interacting with other molecules leads to the unfolding of self-inhibiting domains and activates biological functions (Uversky, 2013). All these examples above show that protein interaction sites play an important role in the transition between disordered protein and ordered protein.

The identification of protein-protein interaction sites is a key to deciphering the functional relationship between proteins and biological processes, which is one of the most important tasks for both experimental and computational approaches. The commonly used experimental methods to pinpoint binding sites in disordered proteins include nuclear magnetic resonance spectroscopy (NMR) and non-equilibrium transient kinetic techniques (Mollica et al., 2016). NMR identifies binding sites through the changes in chemical shifts and residual dipole coupling (RDC) caused by changes in the magnetic environment during binding (Jensen et al., 2011); non-equilibrium transient kinetic technique refers to identifying binding sites by measuring the changes in optical signals that occur during the binding process of disordered proteins (Mollica et al., 2016). Large efforts have also been devoted to gaining a better knowledge of disordered protein interactions by high throughput methodologies through amino acid substitutions, such as binding energetics study in CcdA (Chandra et al., 2022), mutational studies in c-Myb (Giri et al., 2013), ACTR (transcriptional co-activator for thyroid hormone and retinoid receptors) (Dogan et al., 2013), NCBD domain of CBP (CREB binding protein) (Dogan et al., 2013), Hif 1α (hypoxia inducible factor 1α) (Lindström et al., 2018) as well as MazE6 antitoxin (Chandra et al., 2021). These studies greatly contributed to obtaining a thorough understanding of disordered protein interactions.

Besides the experiment methods, there are a series of computational approaches developed for IDP protein interaction sites (IDP-PPIS) prediction such as MoRFpred (Disfani et al., 2012), SLiMPred (Mooney et al., 2012), ANCHOR (Dosztanyi et al., 2009; Mészáros et al., 2009) and SPINE-D (Zhang et al., 2013). With the increasing amount of disordered protein data available, computational approaches to predict IDP-PPIS are becoming more and more important to aid the expensive and time-consuming experiments to annotate the functional properties of disordered proteins. Predictors on IDP-PPIS are mainly designed for predicting several sub-types of disorder binding sites including molecular recognition features (MoRFs), short linear motifs (SLiMs), disordered protein-binding regions (DPBRs), and semi-disordered regions (Katuwawala et al., 2019b). Molecular recognition features (MoRFs) are short disordered fragments involved in state transitions through four types (α-MoRFs, β-MoRFs, ι-MoRFs and complex-MoRFs) during disordered protein binding activities (Mohan et al., 2006). Short linear motifs (SLiMs) are short disordered protein fragments that bind to the structural domains of proteins, consisting of 3–10 amino acid residues, and the disordered binding regions operated by SLiMs and MoRFs are highly overlapping (Mooney et al., 2012; Weatheritt and Gibson, 2012). Disordered protein-binding regions (DPBRs) are more general binding fragments that include not only short binding regions but also longer fragments (Katuwawala et al., 2019b). Semi-disordered regions refer to regions with a 50% probability of being predicted to be disordered regions, and its functional properties can be used to further predict MoRFs (Katuwawala et al., 2019b).

There are several review articles (Katuwawala et al., 2019a; Katuwawala et al., 2019b) on the predictors of IDP-PPIS have been published previously, but most lack systematic descriptions of the factors affecting the performance of the predictors. Moreover, it is time to conclude the latest predictors due to the rapid update of the IDP interaction site predictors. For these purposes, we select 30 predictors of IDP-PPIS published up to January 2022 and provide a comprehensive description of the database, features, and algorithms used in the predictor construction process. This paper gives a detailed overview of the status of the predictors of IDP-PPIS and thus provides new insights and inspiration for the development and application of new computational approaches.

## 2 Overview of the predictors of intrinsically disordered protein-protein interaction sites

**FIGURE 1:**
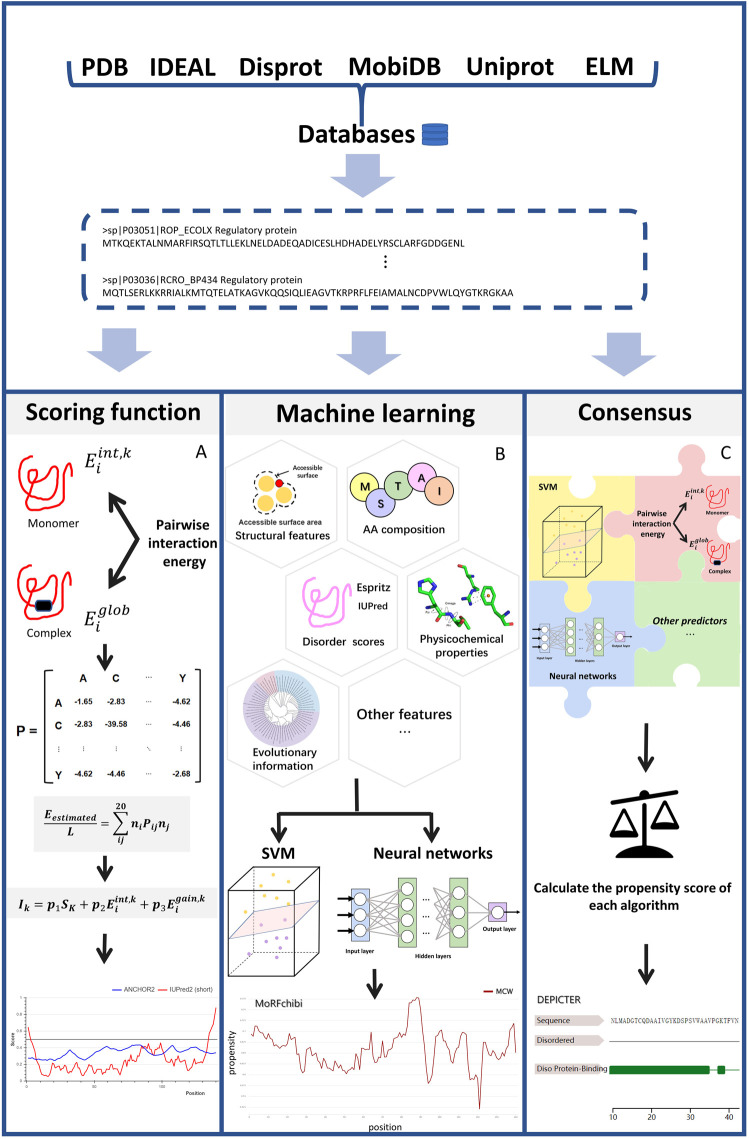
The work flow of each type of methods Figure 1 illustrates main three categories described in this article: **(A)** scoring function-based methods **(B)** machine learning-based methods and **(C)** consensus-based methods. The key steps for each type of methods are depicted in the diagram. Scoring function-based methods in **(A)**, we use ANCHOR work flow to represent how scoring function works. Machine learning-based methods perform the prediction using various types of machine learning models like SVM and Neural Network based on features extracted from different perspectives. Consensus-based methods can predict IDP binding site by weighting different prediction models and combining them optimally. The final results show in **(A)** was processed by (Mészáros et al., 2018). The final results show in **(B)** was processed by (Malhis et al., 2016), The final results show in **(C)** was processed by (Barik et al., 2020).

Figure 1 illustrates the common scheme for developing an IDP-PPIS predictor. Firstly, datasets are curated and selected from large databases and/or published papers. Then, features are extracted from protein sequences using different methods. Then, different algorithms are applied to train and optimize the predictors to output a real-valued amino acid propensity score or binary prediction. Here, we classify the predictors into three categories based on the algorithms used: scoring functions, machine learning-based methods, and consensus-based approaches.

### 2.1 Databases

From Figure 1, the first key aspect of developing the predictor is the selection of a sufficient amount of high-quality data in a standardized format. We present databases widely used in predictors and describes some relevant databases.

The Database of Protein Disorder (DisProt) (Sickmeier et al., 2007) is the first public available database on IDPs and IDRs which includes experimentally annotated information on multiple types of proteins. DisProt is one of the common databases for IDP-PPIS predictors, and the latest version is DisProt 9, covering more than 2000 proteins and nearly 5,000 functional annotations (Quaglia et al., 2021). DisProt is available at https://www.disprot.org/.

Disordered protein regions are often characterized by the missing electron density found by X-ray (DeForte and Uversky, 2016). Several different X-ray experiments are used to examine the same protein to achieve a more stable definition of disordered regions (Monzon et al., 2020). Therefore, The Protein Data Bank (PDB) (Burley et al., 2018), the largest protein 3D structure database is often used for obtaining the contact details of disorder regions in structured proteins. PDB is available at https://www.rcsb.org/.

The missing annotations are related to the inherently disordered protein regions. Several methods implemented UniProt (Apweiler et al., 2010) for disordered prediction, e.g., MobiDB-lite (Bateman et al., 2021). UniProtKB (Apweiler et al., 2010) is a protein database managed by experts, which consists of UniProtKB/Swiss-Prot containing manually annotated data. UniProt (Apweiler et al., 2010) is available at https://www.uniprot.org/.

Intrinsically Disordered proteins with Extensive Annotations and Literature (IDEAL) (Fukuchi et al., 2011) is another commonly used database for disordered protein studies, covering reliable evidence of disordered proteins. In particular, annotations for protein binding regions are helpful for binding sites prediction. There are more than 10,000 non-redundant IDRs in IDEAL as of October 2021. IDEAL (Fukuchi et al., 2013) is available at http://www.ideal.force.cs.is.nagoya-u.ac.jp/IDEAL/.

The Disordered Binding Site (DIBS) (Schad et al., 2017) is a large database storing disordered protein complexes, primarily in combination with ordered proteins. The synthesized information provided on protein interactions is an important resource for studying binding sites. DIBS is available at http://dibs.enzim.ttk.mta.hu/.

The Eukaryotic Linear Motif (ELM) (Dinkel et al., 2011; Kumar et al., 2021) is the first database focused on collecting, storing and providing experimentally confirmed short linear motif (SLiM) information and is an important repository for studying protein-protein interactions of SLiM. ELM is available at http://elm.eu.org.

MobiDB (Piovesan et al., 2020) is a database of IDPs commonly used by predictors, forming four-level annotations of ' CDHP ' (curated, derived, homology, prediction) by connecting other databases and applying various tools. The latest version is MobiDB 4.1, which contains more than 200 million proteins. MobiDB is available at http://mobidb.bio.unipd.it/.

The Structural Classification of Proteins (SCOP) (Andreeva et al., 2019) is a database for storing protein domains. Due to the differences in the evolutionary properties and structural similarity of proteins. Protein domains are divided into various categories. There are six classification levels in SCOP which IUPR is related to IDP. SCOP is available at http://scop.mrc-lmb.cam.ac.uk.

There are some relevant and commonly used databases such as Database of Disordered Protein Prediction (D2P2) and Mutual Folding Induced by Binding (MFIB). D2P2 (Oates et al., 2012) is a database for disorder and SCOP domain prediction and annotation of over 10 million protein sequences by multiple predictors. D2P2 is more commonly used for the study of protein disorder and structural relationships. D2P2 is available at http://d2p2.pro.

MFIB (Fichó et al., 2017) is the database consisting only of complexes formed by intrinsically disordered proteins, and with far more data than other similar datasets, it is an important repository for studying disordered protein interactions. MFIB is available at http://mfib.enzim.ttk.mta.hu/.

### 2.2 Features

The second critical part of constructing predictors is to obtain representative features of disordered protein sequences. Features integrated by the predictor determine the accuracy of distinguishing between IDP protein sites and general amino acid residues. In IDP-PPIS prediction, the most widely used features include amino acid compositions, predicted structural features, disorder scores, physicochemical properties, evolutionary information, and other features. We summarize these features and tools to generate these features in Table 1.

**TABLE 1:** Common features of intrinsically disordered protein-protein interaction sites predictors.

Categories	Features	Tools to calculate	References of the tools
Amino acid composition	Amino acid composition	$\mathrm{composition}\left(i\right)=\frac{n_{i}}{N}$	Wang et al. (2017)
Predicted structural features	Secondary structure	PSIPRED & GOR-I & SPIDER2 & SPOT-1D & Porte	Garnier et al. (1996); Jones, (1999); Pollastri and McLysaght, (2004); Heffernan et al. (2015); Hanson et al. (2018)
	Accessible surface area	SPIDER2 & SPOT-1D & EDTSurf	Xu and Zhang, (2009); Heffernan et al. (2015); Hanson et al. (2018)
	Backbone angle	SPIDER2 & SPOT-1D	Heffernan et al. (2015); Hanson et al. (2018)
	Hemispheric exposure	SPIDER2 & SPOT-1D	Heffernan et al. (2015); Hanson et al. (2018)
	Contact numbers	SPIDER2 & SPOT-1D	Heffernan et al. (2015); Hanson et al. (2018)
	B-factor	ProDy & PROFbval	Schlessinger et al. (2006); Wong and Gsponer, (2019b)
	Structural motifs	BRNN	Mooney et al. (2006)
Disorder scores	Disorder scores	IUPred & Espritz & VSL2 & DISOPRED2 & DISOclust & MFDp	Ward et al. (2004); Dosztanyi et al. (2005); Peng et al. (2006); McGuffin, (2008); Mizianty et al. (2010); Walsh et al. (2011)
Physicochemical properties	Physicochemical properties	AAindex database	Kawashima et al. (2007)
Evolutionary information	Position-Specific Scoring Matrix	PSI-BLAST	Altschul, (1997)
	Bigram feature		
	Hidden Markov Model	HHblits	Remmert et al. (2011)
	Shannon entropy		
Other features	The length and location of IDR		
	Sequence complexity	SEG algorithm	Peng and Kurgan, (2015)

#### 2.2.1 Amino acid composition

Amino acid composition (AAC) is the proportion of a particular amino acid in the whole protein sequence. The amino acid compositions are distinct among IDR, MoRF, flanking regions and other protein regions. It is reported that there are more Ile, Leu, Phe, Tyr, Lys, Arg and Asp enriched and less Ala and Gly comparing with the normal regions. Meanwhile, the flanking region includes more Ala, Gly, Glu, Ser and Thr which turns to promote disorder (Fang et al., 2013; Wang et al., 2017).

AAC could be calculated by the formula (Wang et al., 2017): $\mathrm{composition}\left(i\right)=\frac{n_{i}}{N}$ . i is a particular amino acid; $n_{i}$ is the number of a particular amino acid; N is the total number of amino acids in the protein sequence.

#### 2.2.2 Predicted structural features

The rapid structural transformation of disordered proteins during binding (Uversky and Dunker, 2010) suggests that the predicted structural characteristics of disordered proteins can improve the accuracy of binding site prediction. Commonly used predicted structural features are: secondary structure, structural motif, solvent accessible surface area, contact number, hemispheric exposure, backbone angle, and B-factor.

Molecular recognition feature (MoRF) and short linear motif (SLim) are involved in protein-protein interaction as a secondary structure element (Oldfield et al., 2005; Davey et al., 2012b), including α-helix, β-sheet, curl and other forms. Therefore, using the secondary structure (SS) of protein as a feature can better identify the binding sites. Structural motifs (Efimov, 2017) are folding units formed by the close contact of two or more adjacent secondary structural elements in three-dimensional space, which are often used as a structural feature to predict binding sites.

The biophysical properties of disordered protein change during protein interaction which could be used for IDP-PPIS prediction. For instance, protein-protein interactions are mainly achieved through surface contact, and when the protein state is transformed, the Accessible surface area (ASA) of contact area changes as well, which helps us to identify the binding site (Disfani et al., 2012; Heffernan et al., 2015). Contact numbers (CN) (Yuan, 2005; Heffernan et al., 2015) are also an indicator for measuring protein solvent exposure, which can effectively identify protein contact changes in folding state. Hemisphere exposure (HSE) (Hamelryck, 2005) is a two-dimensional measure for assessing protein solvent exposure that is superior to ASA and CN in terms of computational speed and detection stability. The participation of disordered proteins in protein-protein interactions leads to a shift in the protein backbone (Hanson et al., 2019). The functional realization of disordered proteins is based on their flexible state transitions, and B-factor (temperature factor) is a feature that assesses protein flexibility and helps predict disordered protein binding sites (Disfani et al., 2012; Uversky, 2018).

#### 2.2.3 Disorder scores

Disorder propensity is an important feature for predicting disordered protein binding sites (Dosztányi et al., 2005), and could be predicted by many predictors such as IUPred (Dosztanyi et al., 2005), ESpritz (Walsh et al., 2011), VSL2 (Peng et al., 2006), DISOPRED2 (Ward et al., 2004), DISOclust (McGuffin, 2008), MFDp (Mizianty et al., 2010). IUPred (Dosztanyi et al., 2005) calculates total interacting amino acid pair energy to predict disordered protein regions based on amino acid composition. ESpritz (Walsh et al., 2011) predicts three types of disorder regions (X-ray disorder, DisProt disorder, NMR mobility) based on sequence information using bidirectional recurrent neural networks (BRNN). VSL2 (Peng et al., 2006) predictor consists of VSL2-M1 and VSL2-M2, which solves the problem of heterogeneity in amino acid composition and sequence properties when predicting disordered proteins. DISOPRED2 (Ward et al., 2004) predicts disordered regions using support vector machines based on PSI-BLAST profiles. DISOclust (McGuffin, 2008) predicts disordered proteins by identifying conserved errors in fold recognition models. MFDp (Mizianty et al., 2010) applies three support vector machines to predict different types of disordered regions using multifaceted information.

#### 2.2.4 Physicochemical properties

The structure and function of proteins are affected by the physicochemical properties of amino acids. Disordered proteins are more hydrophobic than ordered proteins, and disordered binding sites can be predicted by an increase in the hydrophobicity (Mészáros et al., 2007). MoRF and linear motifs have more net charge compared to surrounding protein fragments (Fuxreiter et al., 2007). Therefore, the feature of amino acid physicochemical properties is considered in predictors of disordered protein binding sites.

The physicochemical properties of amino acids can be obtained from the AAindex database (Kawashima et al., 2007). The physicochemical and biochemical indexes of amino acids and amino acid pairs in the AAindex database are derived from published literature, which includes three parts amino acid index, substitution matrix and contact potential. In addition, if a protein sequence has similar average amino acid index with MoRF sequence, it suggests that the protein sequence is MoRF (Malhis and Gsponer, 2015). The common physicochemical properties of amino acids include hydrophobicity, polarity, polarizability, charge, aliphatic, aromatics, etc (Fang et al., 2013; Wang et al., 2017).

#### 2.2.5 Evolutionary information

Some studies show that the evolution speed of disordered regions is often faster than other parts of proteins (Brown et al., 2002). Davey et al. (2012a) found that SLiM is more conserved than the surrounding residues and that might be due to protein interactions are highly related to protein functions. Protein evolutionary information is also widely used for protein folding recognition (Lyons et al., 2015), and disordered proteins show protein folding changes during binding. Therefore, many predictors applied protein evolutionary information to identify IDP-PPIS.

Protein evolutionary information is often obtained through position-specific scoring matrix (PSSM), hidden Markov model (HMM) profiles and Information entropy. To improve prediction performance, certain studies use modified PSSM to enhance the sequence conservation signal, such as MFSPSSMpred (Fang et al., 2013) by masking, filtering and smoothing to retain only highly locally conserved information (Fang et al., 2018), obtained highly locally conserved features by amplification.

It is shown that using bigram to extract evolutionary features in natural language processing can reduce redundant features (Sharma et al., 2013), and also extract local evolutionary features for the identification of protein folding process (Lyons et al., 2015). Bigram is also an important feature for identifying IDP-PPIS.

Protein conservation is closely related to its structure and function, and the average Shannon entropy is used as a characteristic of general conservatism in protein. Some studies have used relative entropy to improve the prediction of protein functional sites (Wang and Samudrala, 2006), and Shannon entropy was also applied by (Hanson et al., 2016), to predict IDP-PPIS.

#### 2.2.6 Other features

The length and location of IDRs correlate with general protein functional classes. Lobley et al. (2007) identified short disordered fragments involved in protein interactions in the mid to N-terminal region of GTPase regulatory proteins. FFPred (Minneci et al., 2013) used this feature to predict the biological functions of unknown proteins. Therefore, applying the length and location features of IDRs can improve the prediction accuracy of IDP binding sites.

Compared with the ordered protein, the sequence complexity of the disordered protein is lower (Romero et al., 2000), so the sequence complexity may be an important feature to identify the binding site of IDP. SEG algorithm (Peng and Kurgan, 2015) is mainly used to calculate the complexity of protein sequence.

### 2.3 Algorithms

Algorithm is essential to take the features as input and predict the IDP-PPIS, which is the core element for each predictor. We summarize main information about 30 predictors for IDP-PPIS prediction in Table 2. In this paper, these predictors are classified into three categories according to algorithms: scoring function based, machine-learning based and consensus, and we selectively describe the most representative predictors.

**TABLE 2:** Summary of intrinsically disordered protein-protein interaction sites predictors.

Categories	Years	Predictors	References	Algorithms	Databases	Features	Performance	URL
Scoring function based	2010	retro-MoRFs	Xue et al. (2010b)	Sequence alignment	RNase E and p53 and SRC-3 and SwissProt and PDB	Disorder scores and Sequence similarity	Not Available	Not Available
	2009	ANCHOR	Dosztanyi et al. (2009); Mészáros et al. (2009)	Energy estimation	Disprot and PDB	Pairwise interaction energy	Accuracy 0.67	http://anchor.elte.hu/
	2018	ANCHOR2	Mészáros et al. (2018)	Energy estimation	DisProt and PDB and UniProt and DIBS	Pairwise interaction energy	AUC 0.901	http://iupred2a.elte.hu
Machine-learning based	2012	MoRFpred	Disfani et al. (2012); Oldfield et al. (2018)	SVM	PDB and UniProtKB and Published literature	B-factors and ASA and Disorder scores and Physicochemical properties and PSSM	AUC 0.697	http://biomine.cs.vcu.edu/servers/MoRFpred/
	2013	MFSPSSMpred	Fang et al. (2013)	SVM	PDB and UniProt and Published literature	AAC and PSSM	AUC 0.758	Not Available
	2014	DISOPRED3	Jones and Cozzetto, (2014)	SVM	DisProt and PDB and UniProt	AAC and PSSM and The length and location of IDR	MCC 0.126	http://bioinf.cs.ucl.ac.uk/disopred
	2015	MoRFCHiBi	Malhis and Gsponer, (2015)	SVM	PDB and UniProtKB and Published literature	AAC and Physicochemical properties	AUC 0.770	https://morf.msl.ubc.ca/index.xhtml
	2016	MoRFCHiBiLight	Malhis et al. (2016)	Bayes rule	PDB and UniProtKB and Published literature	Disorder scores and Physicochemical properties	AUC 0.868	http://www.chibi.ubc.ca/faculty/joerg-gsponer/gsponer-lab/software/morf_chibi/
	2016	MoRFCHiBiWeb	Malhis et al. (2016)	Bayes rule	PDB and UniProtKB and Published literature	Disorder scores and Physicochemical properties and PSSM	AUC 0.894	http://www.chibi.ubc.ca/faculty/joerg-gsponer/gsponer-lab/software/morf_chibi/
	2016	fMoRFpred	Yan et al. (2016)	SVM	PDB and UniProtKB and Published literature	AAC and SS and Disorder scores and Physicochemical properties	AUC 0.59–0.67	http://biomine.ece.ualberta.ca/fMoRFpred/
	2016	Predict-MoRFs	Sharma et al. (2016)	SVM	PDB and UniProt	HMM	AUC 0.702	https://github.com/roneshsharma/Predict-MoRFs
	2016	PSSMpred	Fang et al. (2016)	SVM	Disprot and PDB and UniProtKB and ELM	PSSM	AUC 0.758	http://centos.sdutacm.org/fang/SLiMPed.php
	2017	Yu et al	Wang et al. (2017)	SVM	PDB and UniProtKB/Swiss-Prot	AAC and SS and ASA and Physicochemical properties and KNN score	AUC 0.9679	Not Available
	2018	Fang et al	Fang et al. (2018)	SVM	PDB and UniProt	PSSM	AUC 0.713	Not Available
	2018	MoRFPred-plus	Sharma et al. (2018a)	SVM	DisProt and PDB and UniProtKB and Published literature	Physicochemical properties and HMM	AUC 0.821	https://github.com/roneshsharma/MoRFpred-plus/wiki/MoRFpred-plus:-Download
	2007	alpha-MoRFpred	Cheng et al. (2007)	Feed-forward neural networks	PDB and SwissProt	SS and Disorder scores and Physiochemical properties and Shannon’s entropy	Sensitivity 0.87	Not Available
							Specificity 0.87	
							Accuracy 0.87	
	2012	SLiMPred	Mooney et al. (2012)	BRNN	Disprot and PDB and UniProtKB and ELM	SS and Structural motifs and ASA and Disorder scores	AUC 0.69	http://bioware.ucd.ie
	2013	PepBindPred	Khan et al. (2013)	BRNN	ELM and SCOP	SS and Disorder scores and Vina score	AUC 0.75	http://bioware.ucd.ie/pepbindpred
	2013	SPINE-D	Zhang et al. (2013)	Neural-network	DisProt	SS and ASA	MCC 0.15	http://sparks-lab.org
	2016	SPOT-Disorder	Hanson et al. (2016)	LSTM	Disprot and PDB and UniProt	SS and Backbone angles and HSE and CN and ASA and Physicochemical properties and PSSM and Shannon entropy	MCC 0.309	http://sparks-lab.org/server/SPOT-disorder/
	2019	SPOT-Disorder2	Hanson et al. (2019)	LSTM	DisProt and PDB and UniProt and MobiDB	SS and Backbone angles and HSE and CN and ASA and PSSM and HMM	MCC 0.155	https://sparks-lab.org/server/spot-disorder2/
	2021	DeepDISOBind	Zhang et al. (2021)	Multi-task deep neural network	DisProt	SS and RAAPs	AUC 0.771	https://www.csuligroup.com/DeepDISOBind/
	2021	flDPnn	Hu et al. (2021)	RF and Feedforward neural network	DisProt	SS and Disorder scores and PSSM	AUC 0.79	http://biomine.cs.vcu.edu/servers/flDPnn/
	2015	DisoRDPbind	Peng and Kurgan, (2015); Peng et al. (2016)	Logistic regression	DisProt	AAC and SS and Disorder scores and Physiochemical properties and Sequence complexity	AUC 0.62–0.72	http://biomine.ece.ualberta.ca/DisoRDPbind/
	2019	IDRBind	Wong and Gsponer, (2019b)	Gradient boosted trees and CRF	PDB and IDEAL and peptiDB and Docking Benchmark 5 and Published literature	AAC and B-factors and ASA and Physicochemical properties and PSSM	MCC 0.31	https://idrbind.msl.ubc.ca/
Consensus	2018	OPAL	Sharma et al. (2018b)	Integrating predictors	PDB and UniProtKB and Published literature	SS and Backbone angles and HSE and ASA and Physiochemical properties	AUC 0.795–0.870	http://www.alok-ai-lab.com/tools/opal/
	2018	OPAL+	Sharma et al. (2018c)	Integrating predictors	PDB and UniProtKB and Published literature	SS and Backbone angles and HSE and ASA and Physicochemical properties and HMM and Bigram feature vectors	AUC 0.820–0.876	http://www.alok-ai-lab.com/tools/opal_plus/
	2019	Sharma et al	Sharma et al. (2019)	Integrating predictors	PDB and UniProtKB and Published literature	SS and Backbone angles and HSE and CN and ASA and Physicochemical properties	AUC 0.797–0.881	https://github.com/roneshsharma/BMC_Models2018/wiki
	2020	HybridPBRpred	Zhang et al. (2020)	Integrating predictors	DisProt and PDB	AAC and SS and ASA and Disorder scores and Physiochemical properties and HHM and RAAP	AUC 0.795	http://biomine.cs.vcu.edu/servers/hybridPBRpred/
	2020	DEPICTER	Barik et al. (2020)	Integrating predictors	DisProt and PDB	AAC and SS and Disorder scores and Physiochemical properties and Sequence complexity and Pairwise interaction energy	AUC 0.87	http://biomine.cs.vcu.edu/servers/DEPICTER/

#### 2.3.1 Scoring function based

The scoring function method is widely used in the evaluation of protein interaction (Liu and Wang, 2015). Its principle is to obtain the final prediction results by scoring the protein binding ability with various functions (Liu and Wang, 2015). This paper mainly introduces retro-MoRFs based on sequence alignment and ANCHOR series predictors based on paired energy estimation method to predict IDP-PPIS.

##### 2.3.1.1 Sequence alignment

Sequence alignment is a commonly used tool to predict the structural and functional properties of proteins (Edgar and Batzoglou, 2006). Retro-MoRFs (Xue et al., 2010b) predictor used the software package PONDR-RIBS to make sequence alignment by CLUSTALW method (Thompson et al., 1994), and then successfully predicted α-MoRF in RNase E, p53 and SRC-3 by combining PONDR-FIT (Xue et al., 2010a)and PONDR-VLXT (Romero et al., 2000) out-of-order prediction. The predictor innovatively used reverse sequence alignment to identify retro-MoRF.

##### 2.3.1.2 Energy estimation

ANCHOR (Dosztanyi et al., 2009; Mészáros et al., 2009) is a benchmark method in IDP-PPIS prediction. Compared with other predictors, ANCHOR did not include the features of the secondary structure and its combining partners, but still had good predictive performance. The accuracy of ANCHOR reaches 0.67. The principle of the predictor is to predict IDP-PPIS based on the transition of disordered proteins from energy-deficient state to energy-sufficient state during binding. Based on this principle, ANCHOR identified the fragments which were located in the disordered region which could not form enough favorable intra-chain interactions to fold on their own, and might gain stable energy by interacting with globular proteins partners.

The algorithm of the ANCHOR can be expressed as:

$$I_{k}=p_{1}S_{K}+p_{2}E_{i}^{\mathrm{int},k}+p_{3}E_{i}^{\mathrm{gain},k}$$

$I_{k}$ represents the final predicted score for residue k, which is converted to a probability value as the final output. $S_{K}$ corresponds to criterion 1, and the mean IUPred scores of the neighbors with the k -th amino acid in a window range are calculated, so that the disorder trend of the neighborhood with the k -th amino acid is obtained. $E_{i}^{\mathrm{int},k}$ is the possible interaction energy of the k -th residue through forming intrachain contact. $E_{i}^{\mathrm{gain},k}$ is the energy that the residue might gain by interacting with a hypothetical globular protein.

On the basis of ANCHOR (Dosztanyi et al., 2009), ANCHOR2 (Mészáros et al., 2018) increased the energy used to estimate the interaction between spherical protein and local disordered sequence environment. In other words, IDP-PPPS must be exposed to disordered environments and finish the binding process on the surface. ANCHOR2 performs well with AUC up to 0.901.

The new function is defined as follows:

$$S_{k}=\left(E_{\mathrm{gain},k}\left(w_{1}\right)-E_{\mathrm{gain},0}\right)\left(I_{k}\left(w_{2}\right)-I_{0}\right)$$

Here, $S_{k}$ represents the score of the residue k , $E_{\mathrm{gain},k}\left(w_{1}\right)=E_{\mathrm{loc},k}\left(w_{1}\right)-E_{\mathrm{int},k}$ represents the energy calculated only by binding to ordered proteins. $I_{k}\left(w_{2}\right)$ is the average IUPred score of the $w_{2}$ half-window continuous neighborhood of residue k , $E_{\mathrm{gain},0}$ and $I_{0}$ represent the parameters of the minimum energy gain and the minimum average disorder tendency, which make the residues become IDDP-PPIS.

#### 2.3.2 Machine-learning based

From Table 2, we can observe that more than two third of the predictors are based on machine learning. These types of methods usually integrate multiple features derived from the protein sequence information into the model, and use a variety of machine learning algorithms to train the predictors and predict the IDP-PPIS. The prediction results include the binding site propensity score and binary classification prediction. Our collection of machine learning-based predictors mainly uses support vector machines (SVM) (Chang and Lin, 2011) and various types of neural networks (Cheng et al., 2007). In addition, other algorithms such as Logistic regression (Li et al., 1999), gradient boosting trees (Wong and Gsponer, 2019b), conditional random fields (Wong and Gsponer, 2019b) and random forests (RF) (Basu et al., 2017) are also used.

##### 2.3.2.1 Support vector machine

Support vector machine (SVM) (Fan et al., 2008; Chang and Lin, 2011) is a supervised machine learning method, which has been widely used to solve classification and regression problems. Support vector machines usually use kernel functions to solve linear (such as linear kernel) and nonlinear problems (such as Sigmoid function and radial basis function (RBF)). The commonly used SVM algorithms in these predictors are LIBSVM and LIBLINEAR. The data were processed by SVM and a probability value was obtained. When the probability value was greater than 0.5, the amino acid residue was considered as a protein binding site.

MoRFpred (Disfani et al., 2012) is a predictor for identifying different types of MoRF based on protein sequence derived information, using linear kernel support vector machine (SVM) and annotation generated by sequence alignment. MoRFpred chose SVM model with parameter $C=2^{-6}$ , and the prediction is with AUC up to 0.697.

Due to the slow running speed of MoRFpred for large-scale prediction (Yan et al., 2016), developed a fMoRFpred predictor using SVM method to identify MoRF in 2016. fMoRFpred used the data set of MoRFpred, but selected a larger number of feature sets such as predicted disordered region and secondary structure, and the features of a smaller sliding window to improve the performances. Meanwhile, fMoRFpred also used only high-throughput disordered predictors and secondary structure to speed up the computing. The SVM model used by fMoRFpred chose the default parameter C = 5 , and the PPR of fMoRFpred is close to 1, which is better than MoRFpred. In addition, running time analysis shows that fMoRFpred runs faster than MoRFpred.

MFSPSSMpred (Fang et al., 2013) improved the position-specific scoring matrix (PSSM) encoding scheme and extracted protein sequence information from PSSM, and finally applied an SVM model with kernel radial basis function (RBF) to predict MoRF. MFSPSSMpred outperforms other predictors in their paper with the highest AUC of 0.758.

In 2016 (Fang et al., 2016), developed a PSSMpred predictor for predicting the SLiM region. PSSMpred also only used the evolutionary information obtained from the position-specific scoring matrix (PSSM), and applied the SVM model with the kernel of radial basis function (RBF). Its performance is also better than that of other predictors, and obtained the AUC of 0.758.

DISOPRED3 (Jones and Cozzetto, 2014) used amino acid composition, PSSM obtained by PSI-BLAST and the length and location of IDR to apply an SVM classifier with an RBF kernel to predict protein binding sites. DISOPRED3 performs well with an MCC of 0.126.

MoRFCHiBi (Malhis and Gsponer, 2015) trained SVM_S_ and SVM_T_ models of Sigmoid and Radial Basis Function (RBF) Gaussian kernels using the physicochemical properties of amino acids. MoRFCHiBi predicts MoRF by integrating the results of both models with the help of Bayesian rules. It employed SVM_S_ to predict MoRF propensity based on component comparison information and SVM_T_ to predict MoRF propensity based on similarity information. MoRFCHiBi performs better than other predictors with the highest AUC of 0.770 but slower than ANCHOR.

MoRFCHiBiLight (Malhis et al., 2016) used Bayes rules to combine MoRFCHiBi with ESpritz’s (Walsh et al., 2011) disordered prediction results to obtain the final MoRF propensity score. MoRFCHiBiLight performs better than MoRFCHiBi with a maximum AUC of 0.868.

MoRFCHiBiWeb (Malhis et al., 2015; Malhis et al., 2016) predicts MoRF using Bayes rules combining the MoRFCHiBi and MoRF_DC_ predictors (Malhis et al., 2015). MoRF_DC_ used Bayes rules to integrate disorder scores and the conservativeness scores obtained by PSI-BLAST. The AUC of MoRFCHiBiWeb is up to 0.894.

Predict-MoRFs (Sharma et al., 2016) is the first predictor to predict MoRFs using protein sequence evolution information obtained from HMM profiles, and applied SVM with both radial basis function (RBF) and sigmoid kernels to calculate amino acid residue propensity scores. Predict-MoRFs outperformed other predictors with an AUC of 0.702. Since Predict-MoRFs uses the HHblits method (Remmert et al., 2011) to extract the information of the HMM, it runs faster than MoRFpred.

Later in 2018 (Sharma et al., 2018a), improved the Predict-MoRFs and named as MoRFPred-plus. MoRFPred-plus combines HMM profiles and the feature of physicochemical properties of amino acids by applying SVM models with two kernels, radial basis function (RBF) and sigmoid, to obtain final prediction results. The prediction results are better than other predictors, with a maximum AUC of 0.821.

##### 2.3.2.2 Logistic regression

Logistic regression (Li et al., 1999) is a probabilistic classification algorithm, which has the advantages of short running time and high prediction performance, and is widely used in binary classification problems such as predicting protein disorder and ordered protein-protein interaction (Lin et al., 2004). DisoRDPbind (Peng and Kurgan, 2015) used this method to predict IDP-PPIS.

DisoRDPbind (Peng and Kurgan, 2015) input the features extracted from the protein sequences into a logistic regression model to obtain a propensity score for protein residues involved in disordered RNA, DNA, and protein binding, which was then combined with sequence comparison annotations to obtain a final propensity score. Regression coefficients for the input features in the logistic regression model are determined by the ridge. DisoRDPbind performs well with the AUC from 0.62 to 0.72.

##### 2.3.2.3 Gradient boosted trees and conditional random field models

IDRBind (Wong and Gsponer, 2019a) predicted the binding sites of disordered proteins by combining gradient ascending tree and conditional random field model. IDRBind first used XGBoost from R packet (Chen and Guestrin, 2016) to train two classifiers to identify core and edge interface residues by gradient boosting tree method, and then integrated the predicted scores of the two classifiers by conditional random field (CRF) to form the final classification label. IDRBind performs well with an MCC of 0.31.

Gradient boosting tree performs well in solving classification problems, which can be implemented by R package XGBoost (Chen and Guestrin, 2016). Conditional random field (CRF) can be established by EDTSurf and Instant Meshes, which is a different indirect probabilistic graph model, including scoring components and adjacent components. The score component was composed of feature variables (i.e., the results from the gradient boosting tree), factors describing the compatibility of feature variables and label variables, and category deviation related factors. Adjacent components consist of pairwise factors that contain information from adjacent residues.

##### 2.3.2.4 Random forest

The random forest (RF) model (Basu et al., 2017) is widely used in classification problems. The principle is that each decision tree in a random forest makes a judgment on the example to classify it as a positive or negative result, and the result with the higher number of votes is determined as the final result. The random forest model can be obtained through the *Python* package scikit-learn. Random Forest Model is applied by flDPnn (Hu et al., 2021) to predict IDP-PPIS.

FlDPnn (Hu et al., 2021) used multiple machine learning models to extract predicted feature sets about disorder and disorder function, then applied the disorder feature set to train deep feedforward neural networks to better predict disorder, and finally used a random forest model to combine the disorder function features extracted from the machine learning models and the disorder features obtained from the deep feedforward neural networks to predict the protein binding sites of IDP. flDPnn performs better than other predictors with an AUC of 0.79.

##### 2.3.2.5 Neural network

Neural network is widely used in the study of protein-protein interaction. In this paper, we introduce five types of neural network models: Feedforward neural network (Zell, 1994), Bidirectional recurrent neural network (BRNN) (Mooney et al., 2012), Two-hidden layer neural network (Faraggi et al., 2009), Long Short-Term Memory (LSTM) (Hochreiter and Schmidhuber, 1997), and multi-task deep neural network (Zhang et al., 2021).

Feedforward neural networks (Zell, 1994; Sazli, 2006) are artificial neural networks in which information is transmitted unidirectionally from the input layer to the output layer via a hidden layer. They are classified into single-layer and multi-layer feedforward neural networks according to the presence or absence of hidden layers and are trained using a back propagation algorithm. Alpha-MoRFpred (Cheng et al., 2007) applied the feedforward neural network model to predict IDP-PPIS.

Alpha-MoRFpred (Cheng et al., 2007) used conditional probability method to select a representative feature set, and then constructed a feedforward neural network with a hidden layer, which was trained by the supervised learning algorithm in the neural network toolbox of Matlab, and finally predicted the α-MoRF involved in the combination. The sensitivity, specificity and accuracy of alpha-MoRFpred were close to 0.9.

Bidirectional recurrent neural network (BRNN) was applied to predict IDP-PPIS by SLiMPred (Mooney et al., 2012) and PepBindPred (Khan et al., 2013). BRNN (Schuster and Paliwal, 1997) was to obtain sequence information from the opposite direction to the output layer, that is, to predict disordered protein binding sites using the context information of protein sequences. Due to its recursive nature, BRNN had fewer free parameters.

The architecture of BRNN (Mooney et al., 2012; Khan et al., 2013):

$$o_{j}=N^{\left(O\right)}\left(i_{j},h_{j}^{\left(F\right)},h_{j}^{\left(B\right)}\right)$$

$$h_{j}^{\left(F\right)}=N^{\left(F\right)}\left(i_{j},h_{j-1}^{\left(F\right)}\right)$$

$$h_{j}^{\left(B\right)}=N^{\left(B\right)}\left(i_{j},h_{j+1}^{\left(B\right)}\right)$$

j=1,⋯,N

where $i_{j}$ and $o_{j}$ are the input and output of the neural network at position j , respectively. $h_{j}^{\left(F\right)}$ and $h_{j}^{\left(B\right)}$ are the forward and backward chains of hidden vectors with $h_{0}^{\left(F\right)}=h_{N+1}^{\left(B\right)}=0$ **.** $N^{\left(O\right)}$ **,** $N^{\left(F\right)}$ and $N^{\left(B\right)}$ represent the output update, forward update and backward update functions respectively, which are parameterized by three two-layer feedforward neural networks.

SLiMPred (Mooney et al., 2012) applied BRNN to predict SLiMs using information on predicted secondary structure, structural motifs, solvent accessibility, and disorder prediction. The AUC was 0.69. Khan et al. (2013) developed another predictor for SLiMs, PepBindPred, which applied BRNN to predict SLiMs using information on sequence, predicted secondary structure, disorder scores, and Vina score. Adding Vina score improves the predictor performance. PepBindPred performed well with the AUC of 0.75.

SPINE-D (Faraggi et al., 2009; Zhang et al., 2012; Zhang et al., 2013) used predicted torsional angle fluctuations, predicted secondary structure and solvent accessibility as input features to train the neural network to predict IDP-PPIS. The neural network consists of a neural network with two hidden layers and a filtering layer, using a hyperbolic activation function and guided learning techniques. Each hidden layer contains 51 hidden neurons and a bias, and the filter layer contains 11 hidden neurons. SPINE-D performs well with the MCC of 0.15.

Two-hidden layer neural network architecture used by SPINE-D (Faraggi et al., 2009):

Calculation formula of output result of the hidden layers:

$$h_{k}^{1}=f\left(S_{k}^{1}\right)\mathrm{with}\;S_{k}^{1}=\overset{J}{\underset{j=1}{\sum }}w_{\mathrm{jk}}^{1}\cdot x_{j}$$

$$h_{k}^{2}=f\left(S_{l}^{2}\right)\mathrm{with}\;S_{k}^{2}=\overset{K}{\underset{k=1}{\sum }}w_{\mathrm{kl}}^{2}\cdot h_{k}^{1}$$

Where $x_{j}$ represent the input to the neural network, The first hidden layer contains k neurons and the second hidden layer contains l neurons. f(x) is the activation function, $w_{\mathrm{jk}}^{1}$ are the neural network weights that connect the neurons in the input and the first hidden layer, $w_{\mathrm{kl}}^{2}$ are the neural network weights that connect the neurons in the first and second hidden layer.

The training process of a neural network is to compare the output results, $p_{m}$ , with known values to calculate the sum square error E (e.g., ψ angle):

$$E\left(w_{\mathrm{jk}}^{1},w_{\mathrm{kl}}^{2},w_{\mathrm{lm}}^{3}\right)=\frac{1}{2}\overset{M}{\underset{m=1}{\sum }}{\left(\psi_{m}-p_{m}\right)}^{2}$$

Error, E , optimization by steepest gradient descent method:

$${\dot{w}}_{\mathrm{jk}}^{1}=-\eta \frac{\mathrm{\delta E}}{\delta w_{\mathrm{jk}}^{1}}$$

where η is the learning rate.

Both SPOT-Disorder (Hanson et al., 2016) and SPOT-Disorder2 (Hanson et al., 2019) applied Long Short-Term Memory (LSTM) networks (Hochreiter and Schmidhuber, 1997) to predict IDP-PPIS. LSTM networks are a modified recurrent neural network (CNN) capable of solving long time series problems, including single and bidirectional LSTM. The hidden layer contains one or more neurons capable of storing long term memory, and each neuron determines the input, output or forget constant error conveyor (CEC) through a gate function. Long short-term memory (LSTM) networks have been widely used to solve text classification problems (Singh et al., 2022).

SPOT-Disorder (Hanson et al., 2016) used deep bidirectional long-term and short-term memory cyclic neural network to improve prediction performance. The neural network includes bidirectional cyclic neural network (BRNN) composed of three hidden layers. In the first layer, there is cyclic feedforward layer with correction linear unit (ReLU) activation function. The second and third layers are composed of LSTM. The circulation layer contained 200 neurons and bias in each direction, and each neuron in each direction in the LSTM layer contained 200 memory blocks. The model is trained using back-propagation (BPTT) algorithm. Finally, the probability distribution is obtained using the softmax function. The MCC value is 0.309.

The neural network structure of SPOT-Disorder2 (Hanson et al., 2019) consists of IncReSeNet, LSTM and fully connected (FC) layers. IncReSeNet contains three parts: an initial path, a Squeeze-and-Excitation network and a residual connection, each consisting of a residual connection and two convolutional paths with three and one convolutional operations, respectively. SPOT-Disorder2 used similar features as SPOT-Disorder and applied hidden Markov model (HMM) features from HHblits. The MCC value is 0.155.

Based on the information derived from protein sequences, DeepDISOBind (Zhang et al., 2021) applied multi-task deep neural network to accurately predict the binding regions of disordered protein with DNA, RNA and protein. DeepDISOBind included shared layer, nucleic acid binding layer, protein binding layer, DNA binding layer and RNA binding layer. Various sequence feature would be input into a shared layer, which is compose of four different kernel convolutional neural network (CNN) and feedforward neural network (FNN) modules. The shared layer is connected to the protein-binding layer and the nucleic acid-binding layer. The nucleic acid-binding layer is connected to the DNA-binding layer and the RNA-binding layer. The output layer consists of three neurons using sigmoid transfer function, and finally the interaction propensity of disordered protein with RNA, DNA and protein is obtained. For IDP-PPIS prediction, DeepDISOBind outperformed other predictors with an AUC of 0.771.

#### 2.3.3 Consensus

Consensus-based predictor (Fan and Kurgan, 2013) refers to the combination of multiple predictors by using different methods in a weighted manner. The purpose of using consensus predictor is to improve prediction accuracy.

OPAL (Sharma et al., 2018b) is a consensus predictor that combined two predictors, PROMIS (Sharma et al., 2018b) and MoRFCHiBi, to obtain the MoRF propensity score using the simple average method. The MoRFCHiBi predictor is described in detail above. The average method is the sum of the scores of all SVM models divided by the number of models used. The OPAL predictor performs well with an AUC of 0.795–0.870.

Sharma et al. (2018c) also developed the OPAL + predictor in 2018, which is an enhanced version of OPAL. OPAL + combined four independent SVM models with radial basis function (RBF) kernel for different length amino acid residues with MoRFpred-plus and MoRFCHiBi to obtain the final MoRF propensity score by average method. OPAL + performed better than other predictors with AUC of 0.820–0.876.

Another consensus-based predictor was also constructed by Sharma et al. (2019). Using the structural information obtained from the protein sequence, two independent SVM models with radial basis function (RBF) as the kernel were used to predict the MoRF located in the terminal and in the middle, respectively. Combined with the prediction scores of the two models, the final MoRF propensity score was obtained with AUCs 0.729–0.864. Then, the predictor was combined with MoRFpred-plus, PROMIS and MoRFCHiBi to form a consensus predictor that can obtain sequence information from different aspects and combine different algorithms. The final MoRF propensity score was obtained by averaging the scores of each predictor. The consensus predictor performs better than other predictors with AUC of 0.797–0.881.

DEPICTER (Barik et al., 2020) designed consensus predictors for predicting protein disorder and protein-binding IDR, respectively, and further improved the protein-binding IDR prediction performance by the disorder consensus predictor. The consensus predictor for IDR-PPIS combined three common predictors DisoRDPbind, ANCHOR2, and fMoRFpred. DEPICTER selected 54 features and applied four machine learning methods, Logistic Regression, Parsimonious Bayes, Random Forest, and Extreme Gradient Boosting Tree, to develop the consensus predictor. The prediction results obtained by DisoRDPbind, ANCHOR2 and fMoRFpred were transformed into feature vectors to be input to the consensus predictor to obtain new disordered proteins combining predicted propensity real values and dichotomous propensity. DEPICTER selected the best performing consensus predictor relying on extreme gradient boosting tree construction for testing, which outperformed the independent predictor with an AUC of 0.87.

The most recent consensus predictor HybridPBRpred (Zhang et al., 2020) combined the predictions of DisoRDPbind trained on disordered annotated data and the predictor SCRIBER trained on structured data to predict different types of protein binding residues. HybridPBRpred first normalized the scores of each predictor to [-1, 1], and for binary prediction, the prediction is protein binding residue score >0 otherwise score <0. Then, the final score was obtained in the following way: if at least one predictor predicts the residue to be a protein-binding residue, the higher score is chosen as the final score; if both predictors predict a non-protein-binding residue, the average of the two scores is used as the final score. The consensus predictor HybridPBRpred has improved IDP-PPIS prediction compared to non-consensus predictors with an AUC of 0.795.

## 3 Discussion

Disorder proteins lack stable structures but have many important functions through protein-protein interactions. There are a large number of studies have focused on identifying the protein binding sites of disordered proteins which will provide better functional annotations of disorder proteins. We scanned the literature since the publication of alpha-MoRFpred in 2007 and found that there has been a sharp increase in protein-binding IDR predictors, with 6–10 new predictors published every 3 years. These predictors continuously improve the prediction performance by applying different algorithms, screening more representative features or combining multiple models, and so on. The AUC is increased from 0.6 to 0.9. However, there might be still some limitations for current methods which can be improved from several directions.

We found that the existing predictors mainly curated the datasets from two large databases Disprot and PDB, especially there are many predictors on MoRF using the high-quality dataset from Disfani et al. Since this dataset contains a large number of immune-related proteins (Fang et al., 2013), the trained predictors might suffer from bias problems and neglect some potential binding sites.

In addition, most of the existing predictors are trained only for disordered protein data, but HybridPBRpred expands the benchmark dataset by combining predictors trained from structured protein datasets, allowing the predictors to improve the prediction performance in terms of protein binding sites for disordered proteins. There might be some common properties shared by different types of protein interfaces (Hou et al., 2017; Hou et al., 2019). Therefore, constructing benchmark datasets by expanding database sources such as from D2P2, MFIB, etc., and using more comprehensive datasets that include not only disordered protein data but also ordered protein data, could improve the performance of predictors in the future.

The existing predictors mainly focus on short binding regions such as MoRF (Fang et al., 2018) and SLiMs (Mooney et al., 2012), but there are still non-MoRF and non-SLiMs binding regions in disordered proteins. Therefore, predicting long disordered binding regions is an important hotspot in future development. In recent years, more and more predictors have improved the prediction performance by combining various features or algorithms. However, this approach easily causes high-dimensional feature space and the algorithm complexity and also reduce the computing efficiency (Malhis et al., 2016).

Salt bridges involved in disordered proteins have not yet been considered as a feature in all the predictors summarized. When disordered proteins participate in protein-protein interactions, ionic bonds are formed between oppositely charged amino acid side-chains, i.e., salt bridges. Studies (Basu and Biswas, 2018; Roy et al., 2022) have found that the formation of salt bridges contributes to the generation of local rigid structure of IDP, and triggers the disordered to ordered transition of IDP. For example, α -synuclein binds with tubulin to form an inter-chain salt bridge and mediate the transformation of protein conformation (Basu and Biswas, 2018). The arginines of FLCS_Spike_ and the anions of Furin form dynamically interchangeable and durable salt bridge networks at the Spike-Furin binding interface, which triggers the transition from disorder to order (Roy et al., 2022). Therefore, we believe that the performance of the protein-binding sites predictor can be further improved by adding the feature of salt bridges in disordered proteins.

Since disordered proteins lack stable tertiary structures, most existing predictors are developed based on sequences, but disordered proteins still retain structural conformational properties such as secondary structure features, and it has been pointed out that transient secondary structure pre-structured motifs (PreSMos) (Kim and Han, 2021) exist in intrinsic disordered proteins and are involved in the development of various diseases by binding to corresponding targets. Furthermore, with the rapid development of structure prediction technology in the protein field, disorder predictors constructed based on AlphaFold2 (Wilson et al., 2022) structures were found to have potentials to identify disordered regions. Alphafold2 and RoseTTAFold have been successfully used to predict the structure of protein complexes (Bryant et al., 2022). The PLDDT value of Alphafold2 is commonly used to identify the structure of protein complex and the disordered regions. Tsaban et al. (2022) found that Alphafold2 recognized SLiM in the peptide-protein complex and also correctly characterized the conformational change upon protein binding. Akdel et al. (2021) proved that Alphafold2 was able to successfully predict the structure of complexes involving disordered proteins. Therefore, the construction of structure-based predictors may further enhance the protein binding site prediction performance of disordered proteins. In addition, inspired by HybridPBRpred, we can also improve the existing structure-based predictors to develop more comprehensive protein binding site predictors.

## 4 Conclusion

In this paper, we collected 30 predictors related to IDP-PPIS published up to January 2022, and described them in terms of three key aspects of the predictor construction process: databases, features, and algorithms. By summarizing the advantages and disadvantages of the existing predictors, we believe that the development of more comprehensive protein-binding site predictors by expanding the data sources, applying the features related to structural changes and binding to ordered protein-binding site predictors may further improve the performance of IDP-PPIS in the near future. In addition, since disordered proteins are involved in a variety of important physiological and biochemical processes using protein-protein interactions in various organisms, we also hope our review will help researchers to gain new ideas for solving various disease problems mediated by disordered proteins.
